# Lipidomic Profiles of Lipid Biosynthesis in Oil Palm during Fruit Development

**DOI:** 10.3390/metabo13060727

**Published:** 2023-06-06

**Authors:** Jerome Jeyakumar John Martin, Qiufei Wu, Meili Feng, Rui Li, Lixia Zhou, Shuyan Zhang, Cheng Yang, Hongxing Cao

**Affiliations:** Coconut Research Institute, Chinese Academy of Tropical Agricultural Sciences/Hainan Key Laboratory of Tropical Oil Crops Biology, Wenchang 571339, China; jeromejeyakumarj@gmail.com (J.J.J.M.); qfi_wu@catas.cn (Q.W.); fengml2002@catas.cn (M.F.); lirui@catas.cn (R.L.); lxzhou@catas.cn (L.Z.); yc990814@webmail.hzau.edu.cn (C.Y.)

**Keywords:** oil palm, metabolome, lipidomics, LC-MS, fatty acids, glycolipids, triacylglycerol

## Abstract

The fruit of the oil palm (*Elaeis guineensis Jacq*.) has fleshy mesocarpic tissue rich in lipids. This edible vegetable oil is economically and nutritionally significant across the world. The core concepts of oil biosynthesis in oil palms remain to be researched as the knowledge of oil biosynthesis in plants improves. In this study, we utilized a metabolite approach and mass spectral analysis to characterize metabolite changes and identify the sequences of protein accumulation during the physiological processes that regulate oil synthesis during oil palm fruit ripening. Here, we performed a comprehensive lipidomic data analysis in order to understand the role of lipid metabolism in oil biosynthesis mechanisms. The experimental materials were collected from the mesocarp of oil palm (Tenera) at 95 days (early accumulation of fatty acid, first stage), 125 days (rapid growth of fatty acid accumulation, second stage), and 185 days (stable period of fatty acid accumulation, third stage) after pollination. To gain a clear understanding of the lipid changes that occurred during the growth of the oil palm, the metabolome data were found using principal component analysis (PCA). Furthermore, the accumulations of diacylglycerols, ceramides, phosphatidylethanolamine, and phosphatidic acid varied between the developmental stages. Differentially expressed lipids were successfully identified and functionally classified using KEGG analysis. Proteins related to the metabolic pathway, glycerolipid metabolism, and glycerphospholipid metabolism were the most significantly changed proteins during fruit development. In this study, LC-MS analysis and evaluation of the lipid profile in different stages of oil palm were performed to gain insight into the regulatory mechanisms that enhance fruit quality and govern differences in lipid composition and biosynthesis.

## 1. Introduction

Oil palm (*Elaeis guineensis Jacq*.), which is a perennial monocotyledon crop [1], is the highest-oil-yielding plant, with a predicted global demand of 240 million tons by 2050 [2]. The fruit pericarp is composed of three layers: the exocarp, mesocarp, and endocarp. The endocarp is a hard shell enclosing the kernel, which contains a small embryo and a large endosperm. Oil palms produce 10% kernel oil and 90% mesocarp oil. Palm oil has almost equal proportions of saturated and unsaturated fatty acids. The primary fatty acids in palm oil are myristic, palmitic, stearic, oleic, and linoleic acids [1,3,4].

Lipids are essential components of biological membranes, protecting cells from external stimuli and serving as hormonal precursors, signaling molecules, and energy sources [5]. Plant survival depends on the dynamic responses of their membrane bilayer, which protects them from biotic and abiotic stresses [6]. Traditionally, lipid nutrition has concentrated on the amount of fatty acids found in complex glycerol and glycerophospholipids. Palm oil lipids consist of TAGs and DAGs, with a low abundance of other lipid classes, such as phospholipids and galactolipids. These low-abundance lipids are key intermediates in lipid biosynthesis [7]. TAGs attract other fat- or oil-soluble components during oil extraction. Palm oil includes trace contaminants and metabolites from TG and lipolytic products. Monoacylglycerols (MGs), diacylglycerols (DGs), and free fatty acids (FFAs) are examples of cellular components [8]. Fatty acids can be beneficial or detrimental, depending on the amount and type consumed. High saturated fatty acid intakes can lead to obesity and chronic diseases, while low intakes can cause hemorrhagic stroke. Increased omega-3 polyunsaturated fatty acid (PUFA) intakes can reduce the risk of cardiovascular diseases. Lipidomics allows for the quantification of complex lipids in the oil. Lipidomic endeavors aim to understand the function and interactions of specific lipids in cell signaling and biochemical pathways. Lipid biosynthesis and accumulation in plants is a complex process involving de novo synthesis, desaturation, assembly, and formation of oil bodies [9]. A lipidomics platform has been developed that covers 300 molecular lipid species from Arabidopsis leaves [10], but some lipid classes that exert regulatory functions are difficult to incorporate due to their low abundance and structural characteristics. Studies were published on the lipid composition of the oil palm mesocarp and kernel during ripening, including the determination of the total fatty acid content and main polar lipids, as well as HPLC analyses of molecular species [11]. Analysis of fatty acid at position sn-2 of TAGs by pancreatic digestion of whole palm oil indicates that 18:1 and 18:2 are predominant. According to Tranbarger et al. [12], unique transcriptional programs control the synthesis of ER-based TAG and plastidial FA, respectively. On the other hand, Ramli et al. [13] showed that WRINKLED (WRI1) and AP2 regulate FA synthesis, indicating a key influence over storage oil synthesis. This research aimed to explore the networks that are correlated with oil synthesis in order to provide a better theoretical foundation for the molecular improvement and genetic breeding of oil palm. It used a highly selective analysis to detect specific precursor–fragment pairs, enabling the development of a targeted lipidomics platform.

## 2. Materials and Methods

### 2.1. Plant Materials

Oil palms were planted in the oil palm research base of the Coconut Research Institute of the Chinese Academy of Tropical Agricultural Sciences (19°33′ N, 110°47′ E; Wenchang; Hainan Province). Fresh fruits (flesh and nucleolus of the female flowers) at the different ripening stages were collected on the 95th, 125th, and 185th days after pollination. For the RNA extractions and chemical composition analysis, samples were collected and frozen immediately in liquid nitrogen and stored in a refrigerator at −80 °C. Three biological replicates were prepared using pulp and nucleolus samples of oil palm at each development stage.

### 2.2. Sample Preparation and Extraction

Lipids were extracted (MTBE) using a solvent system of methanol and methyl tert-butyl ether. More specifically, the sample was thawed before being added to the 2 mL centrifuge tube along with 1 steel bead and 20 mg of dry material (internal diameter of about 4 mm). Then, 1 mL of the lipid extract (MTBE:MeOH = 3:1) was added and the contents were vortexed for 30 min. After adding 300 µL of ultrapure water and vortexing for 1 min, the sample was kept at 4 °C for 10. After centrifugation at 12,000 rpm for 3 min at 4 °C, 400 µL of supernatant was transferred to a 1.5 mL centrifuge tube and concentrated to absolute dryness at 20 °C. After vortexing for 3 min and centrifugation at 12,000 rpm for 3 min at 4 °C, 200 L of lipid complex solution (ACN:IPA = 1:1) was added to redissolve. Finally, 120 L of a reconstituted solution was collected for LC-MS/MS analysis.

### 2.3. RNA Extraction and Transcriptomic Analysis

Total RNA from Tenera was extracted using a total RNA Kit and the RNA quality was assessed using agarose gel electrophoresis, a Qubit 2.0 fluorescent agent, and an Agilent Bioanalyzer. RNA-Seq libraries were prepared and sequenced on an Illumina Hiseq2500 platform. The gene expression level was normalized by calculating the fragments per kilobase of transcript per million fragments mapped (FPKM). Deseq2 software version 1.2.0 was used to analyze differentially expressed genes in the flesh and nucleolus at the same developmental stage. KEGG pathway enrichment analysis was performed for differentially expressed genes (DEGs) using R software with a corrected *p*-value < 0.05.

### 2.4. Lipidomic Analysis Using Liquid Chromatography–Mass Spectrometry

Lipids were chromatographically separated using ExionLC ultra-performance liquid chromatography in tandem with a SCIEX QTRAP 6500+ mass spectrometer, and the chromatographic column was a Thermo AccucoreC30 column (2.6 μm, 2.1 mm × 100 mm i.d.). The mobile phases were as follows: A—acetonitrile/water (60/40 *v*/*v*, 0.1% formic acid, 10 mmol/L ammonium formate); B—acetonitrile/isopropanol (10/90, *V*/*V*) (containing 0.1% formic acid and 10 mmol/L ammonium formate). The solvent gradient program was as follows: A/B ratio was 80:20, *V*/*V* at 0 min, 70:30 *V*/*V* at 2.0 min, 40:60 *V*/*V* at 4 min, 15:85 *V*/*V* at 9 min, 10:90 *V*/*V* at 14 min, 5:95 *V*/*V* at 15.5 min, 5:95 *V*/*V* at 17.3 min, 80:20 *V*/*V* at 17.3 min, 80:20 *V*/*V* at 20 min. The flow rate was 0.35 mL/min, the temperature was 45 °C, and the injection volume was 2 μL. The effluent was alternatively connected to an ESI triple quadrupole-linear ion trap (QTRAP)-MS.

### 2.5. Cluster Analyses

By using unit variance scaling (UV), the lipid content data were standardized, and the R software’s heatmap package was utilized to generate heatmaps. Hierarchical clustering analyses (HCAs) were used to examine the lipid accumulation trends among different samples.

## 3. Results

### 3.1. Data Pre-Processing and Quality Control (QC) of the Lipidomics

**Figure 1:**
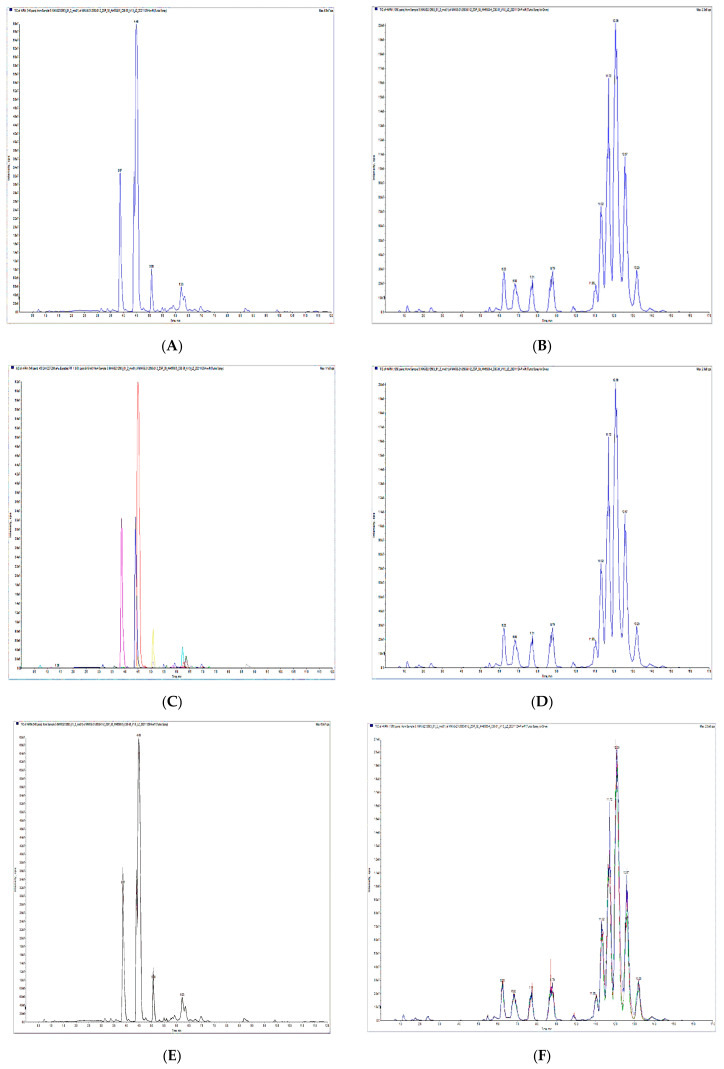

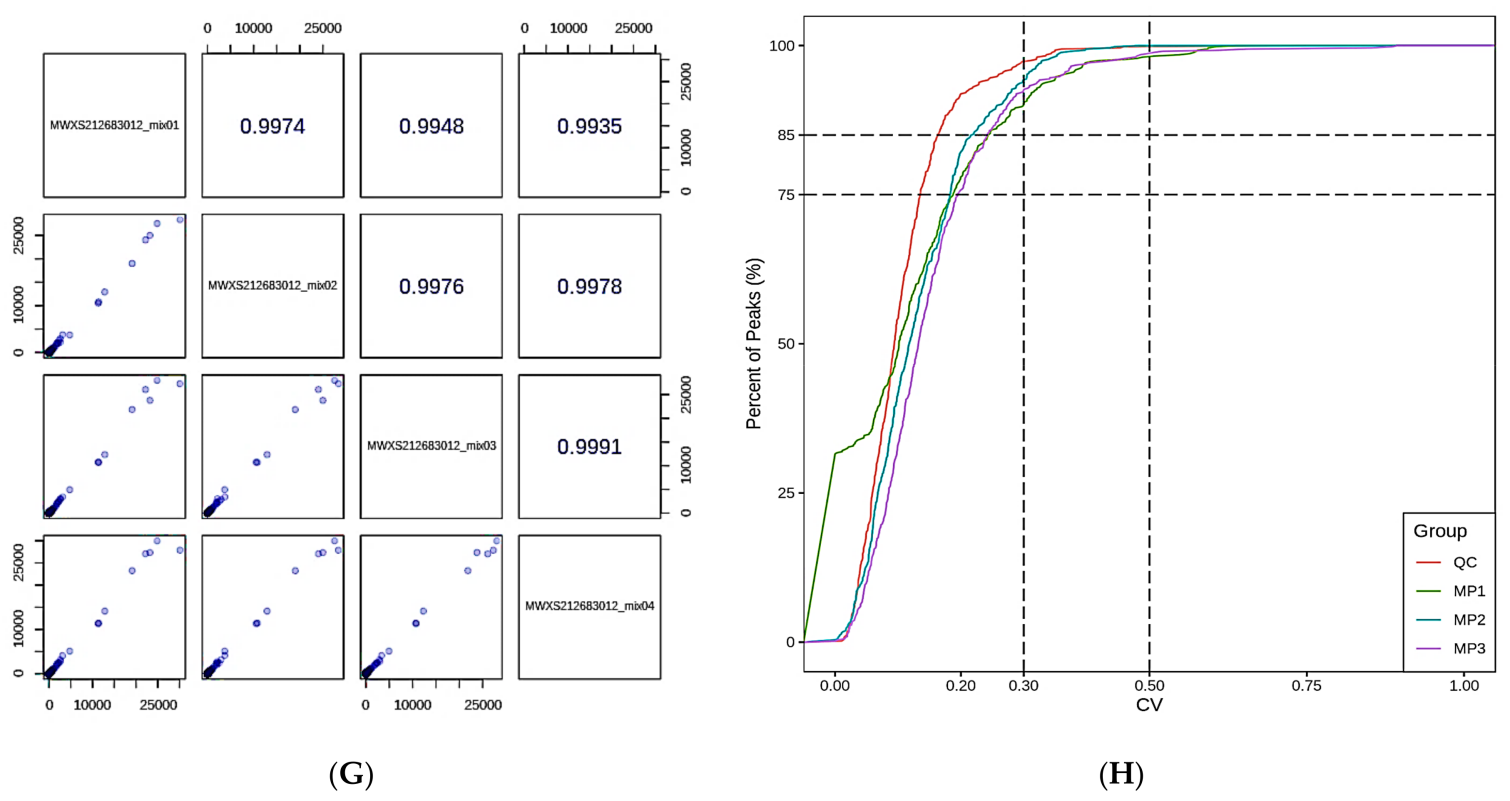
(**A**,**B**) TIC of a mixed QC sample in negative and positive modes, respectively. (**C**,**D**) The MRM of the mixed QC sample in negative and positive modes, respectively. (**E**,**F**) TIC spectra overlapped in negative and positive modes, respectively. (**G**) Schematic representation of the correlation spectra of the QC samples. (**H**) The CV of the QC samples.

Mass spectrometry software is used in mass spectrometry to collect, analyze, or present data. The total ion chromatogram (TIC) is shown in (Figure 1A: negative mode; Figure 1B: positive mode). The intensities of all ion abundances in the mass spectrum were combined in the TIC; therefore, the distinctive power of MS was not exploited. The chromatogram shows several peaks corresponding to different chemicals (Figure 1C: negative mode; Figure 1D: positive mode), with the horizontal axis representing the retention time and the vertical axis representing response intensity. According to the lipid database, mass spectrometry was used to identify lipid metabolites, which were calibrated to ensure accurate quantification. The overlapped total ion chromatogram (TIC) of the QC samples was examined using instrument stability (Figure 1E and Figure 1F show the negative and positive modes, respectively). The stability of a mass spectrometer was found to be relatively constant, allowing the signal to remain stable throughout the analysis process. A Pearson correlation analysis (QC) showed that the testing technique was consistent (Figure 1G), and the coefficient of variation (CV) could be used to measure data dispersion. The CV results revealed that the proportion of compounds in the QC samples with CV values less than 0.5 was more than 85%, and those with CV values less than 0.3 were greater than 75%, exhibiting the high reliability of the experimental data (Figure 1H).

### 3.2. Principal Component Analysis and Cluster Analysis of the Lipidomics

**Figure 2:**
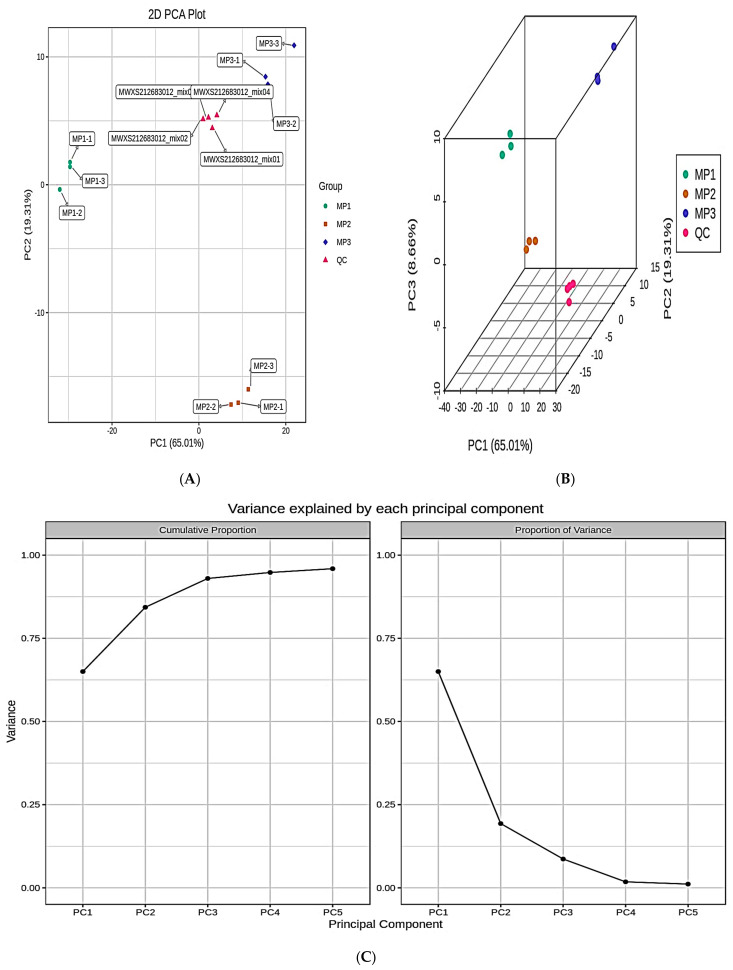

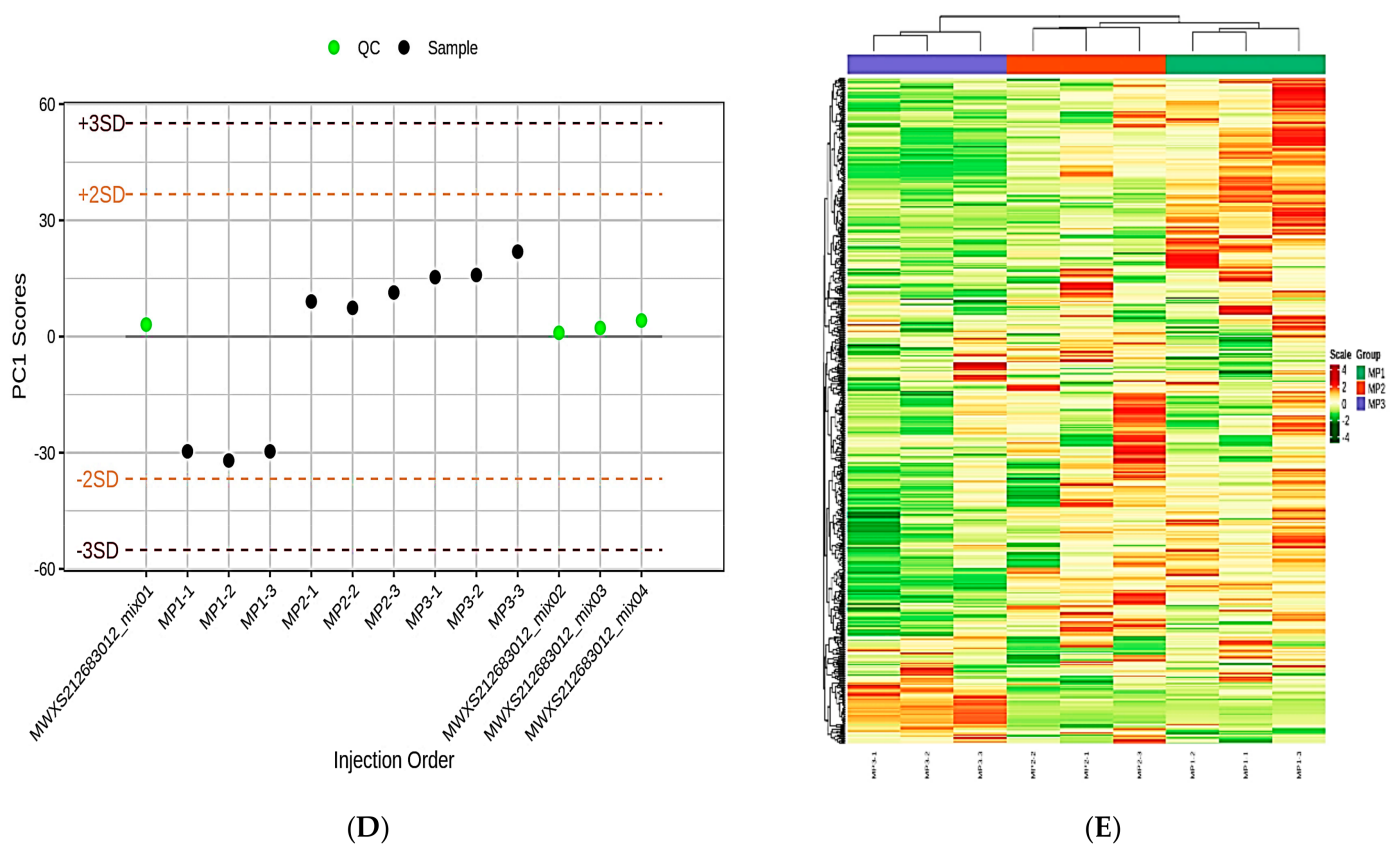
(**A**) The two-dimensional graphic representation of the PCA findings for the entire dataset. (**B**) The 3D representations of the results of the PCA for all samples. (**C**) The plot represents the variance explained by different principal components. (**D**) The vertical coordinates represent the values of PC1, the horizontal coordinates are the sequence of sample tests, and the yellow and red lines indicate the mean plus or minus 2 and 3 standard deviation ranges, respectively. The QC samples are represented by green dots, while the test samples are represented by black dots. (**E**) Cluster analysis results.

The PCA of all samples (including QC samples) revealed a tendency toward separation for lipid metabolites across the MP1, MP2, and MP3 groups (Figure 2A–C). The PCA plot’s horizontal coordinates reflect the first principle component, the vertical coordinates represent the second principal component, and the percentage represents the principal component’s contribution to the sample variance. Each dot on the plot represents a sample. PC1 of the QC samples was within plus or minus 3 standard deviations (SD) on the principal component univariate statistical process control plot, indicating that the instrument’s condition was stable (Figure 2D). The polar difference method was used to standardize the lipid metabolite content. After normalization, R software was used to perform a hierarchical cluster analysis (HCA) to examine the accumulation pattern of metabolites among the samples obtained (Figure 2E).

### 3.3. Lipid Metabolites Composition and Changes in Subclass Content

**Figure 3:**
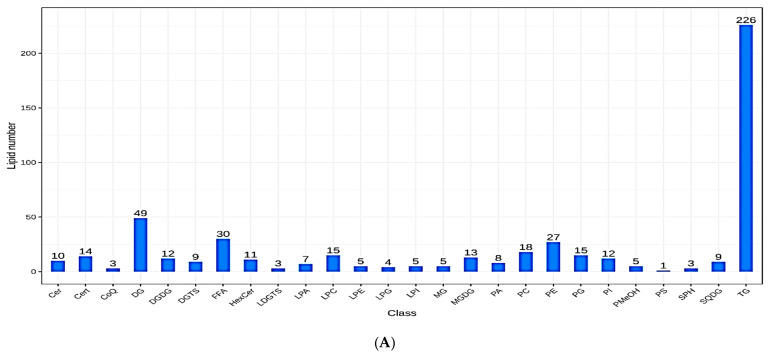

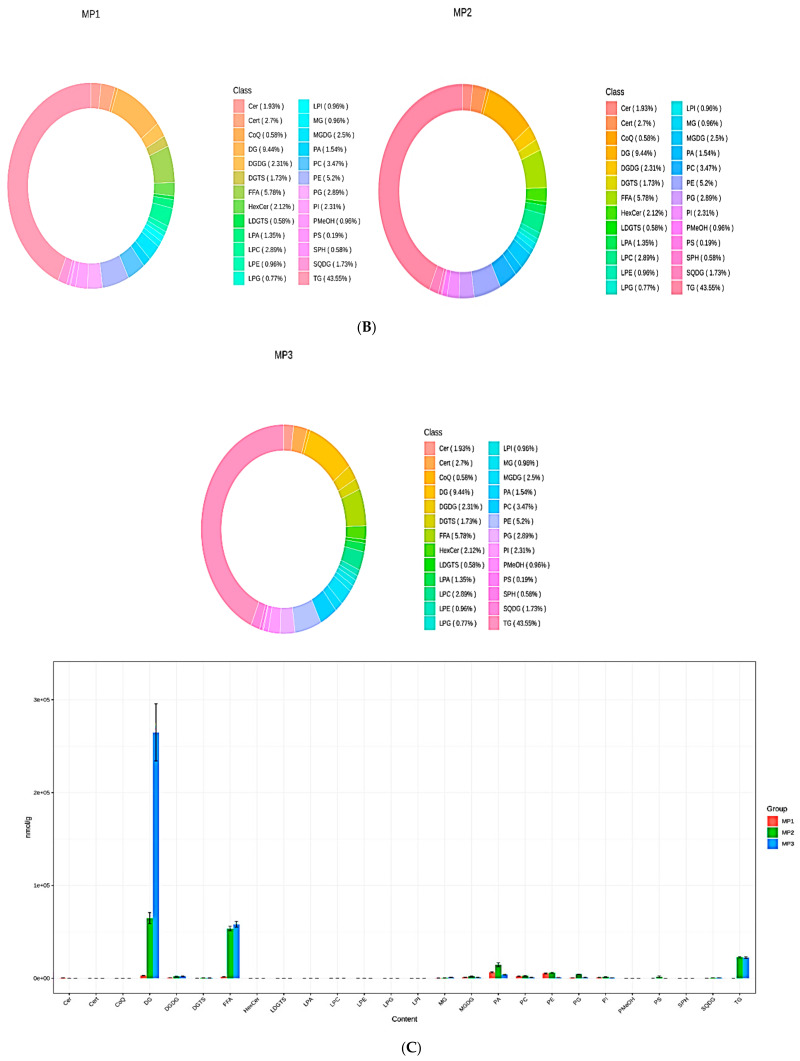

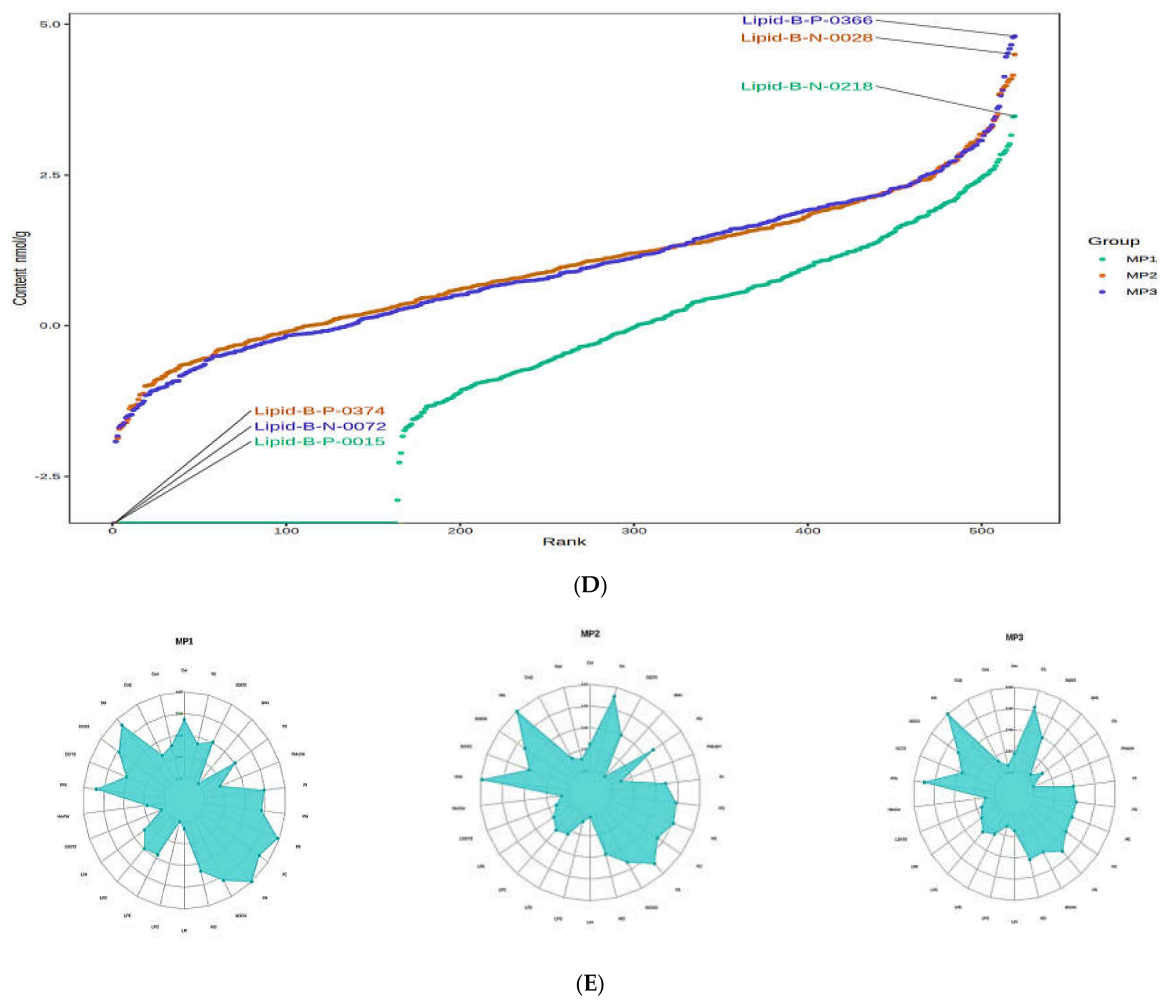
(**A**) Histogram showing the lipid subclasses. (**B**) The ring diagram shows the composition of the lipid subclasses. (**C**) The histogram of changes in lipid subclass content among the groups. (**D**) The dynamic distribution of the lipid content. (**E**) Radar charts showing the lipid metabolite contents.

The statistics of the recognized lipid subclasses with the proportions and amount of lipid metabolites observed in each subclass are presented in Figure 3A,B; meanwhile, Figure 3C shows the differences in lipid subclass composition between MP1, MP2, and MP3. The dynamic lipid content distribution showed variations in lipid content over the spectrum, as well as the lowest and highest lipid metabolites in the groups (MP1, MP2, and MP3) (Figure 3D). The radar plots revealed patterns in the variations in the lipid metabolite concentrations between the groups (Figure 3E).

### 3.4. Discriminant Analysis of the Lipid Metabolites Using Subgroup Principal Component Analysis and Orthogonal Partial Least Squares

**Figure 4:**
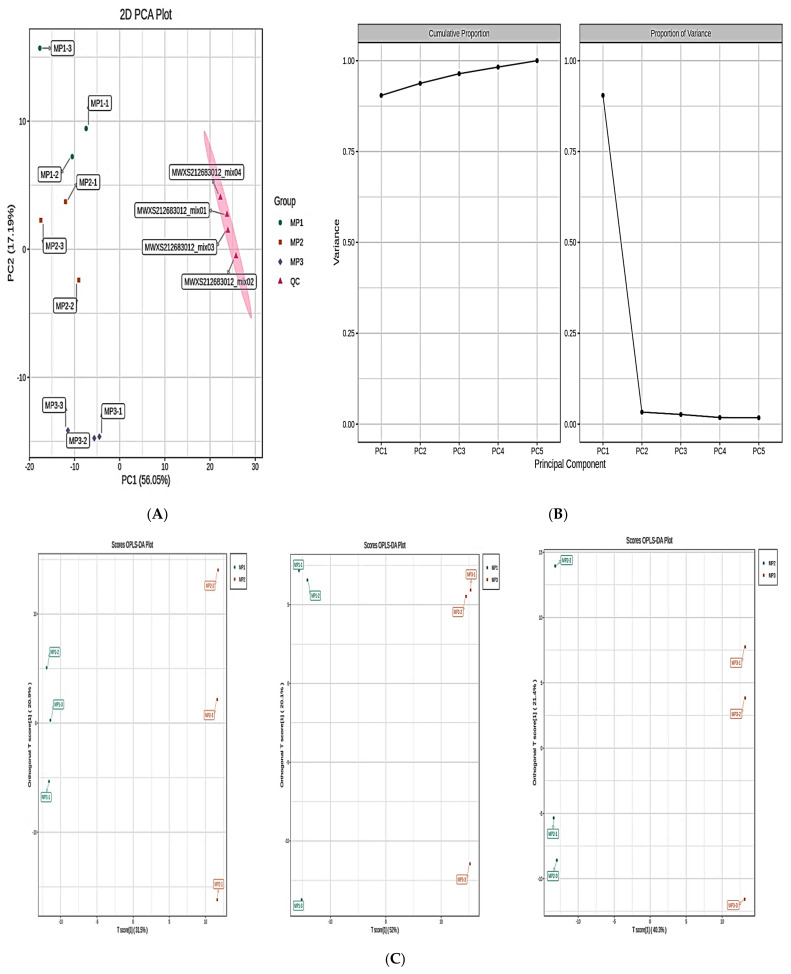

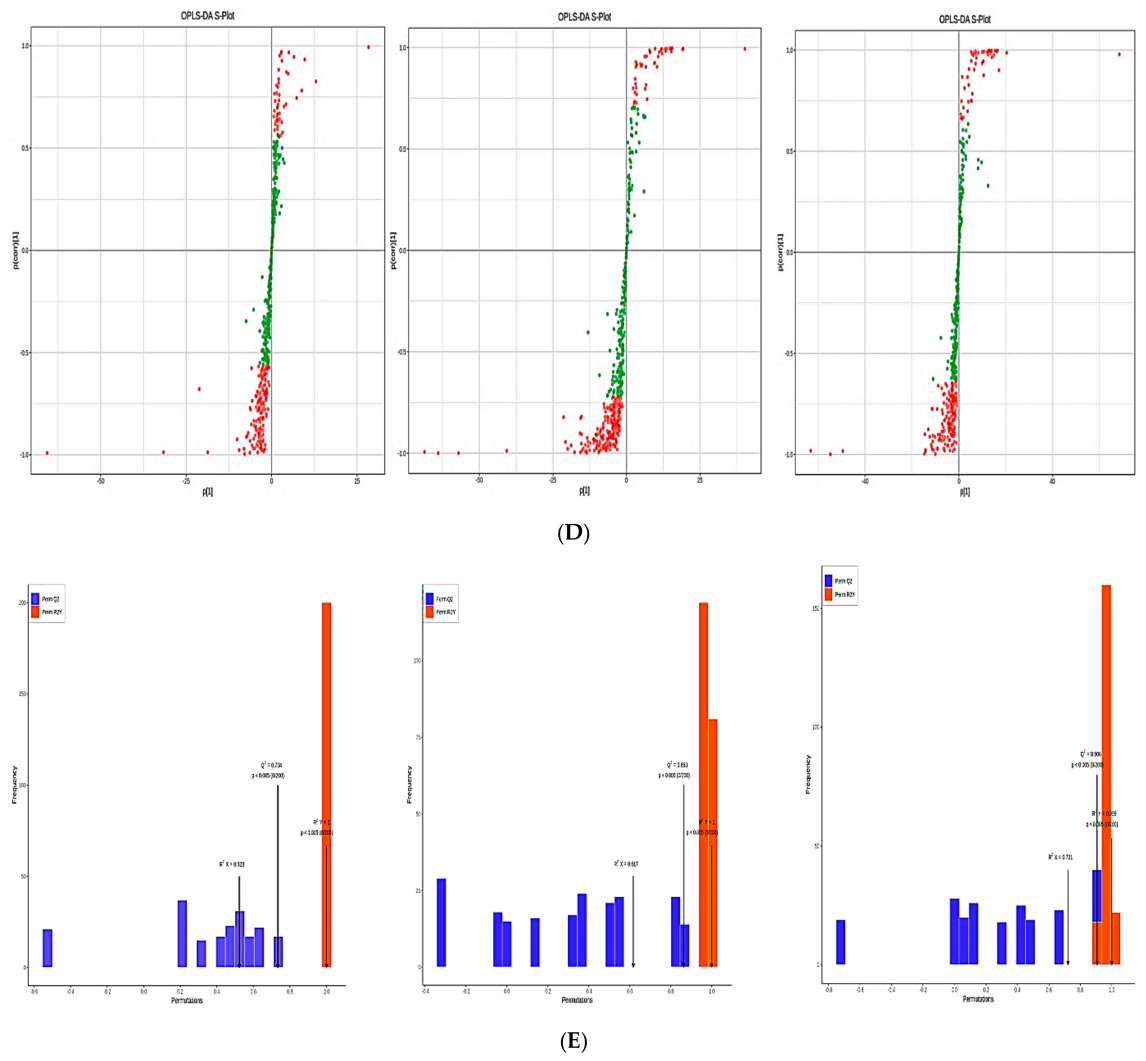
(**A**) Two-dimensional representations of the subgroup PCA. (**B**) The variation explained by each principal component plot. (**C**) OPLS_DA score map. (**D**) OPLS_DA S-plot. (**E**) The permutation test.

Figure 4 displays the subgroup PCA. The confidence intervals of the PCA plots are shown by the ellipse in Figure 4A, where the horizontal coordinate represents PC1 and the vertical coordinate represents PC2. The MP1, MP2, and MP3 groups showed a trend of separation in both dimensions (Figure 4A), which suggests a substantial variation in lipid metabolites between the groups, whereas Figure 4B depicts the explainable variations of the top five main components listed. The vertical coordinates represent the explainable variances, while the horizontal coordinates represent the principal components. Moreover, the right panel displays the explainable variances of each main component, whereas the left panel displays the cumulative explainable variances. An OPLS-DA score plot was used to depict the raw lipidomic data, revealing a clear differentiation between the different stages (MP1, MP2, MP3) (Figure 4C). The red and green dots in the Figure 4D OPLS-DA S-plot represent the metabolites near the upper right and lower left corners, indicating a differential expression (VIP ≥ 1 and VIP *<* 1). The results from the subsequent permutation tests showed that R2X = 0.808, R2Y = 1, and Q2 = 0.983 (Figure 4E), showing that the plots functioned uniformly in terms of predictive performance.

### 3.5. Identification of the Lipid Metabolites

**Figure 5:**
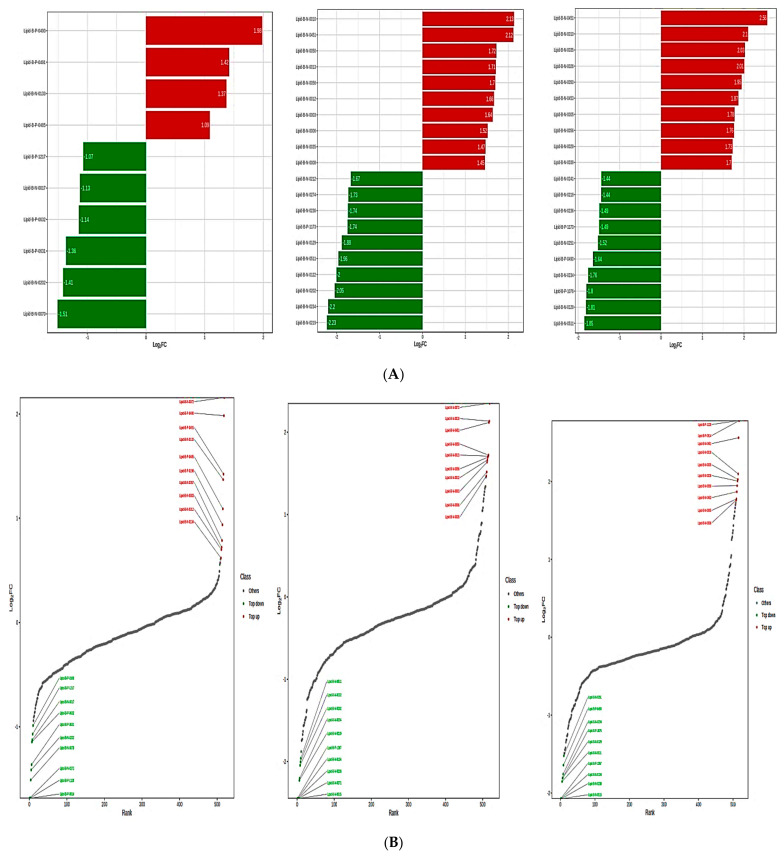

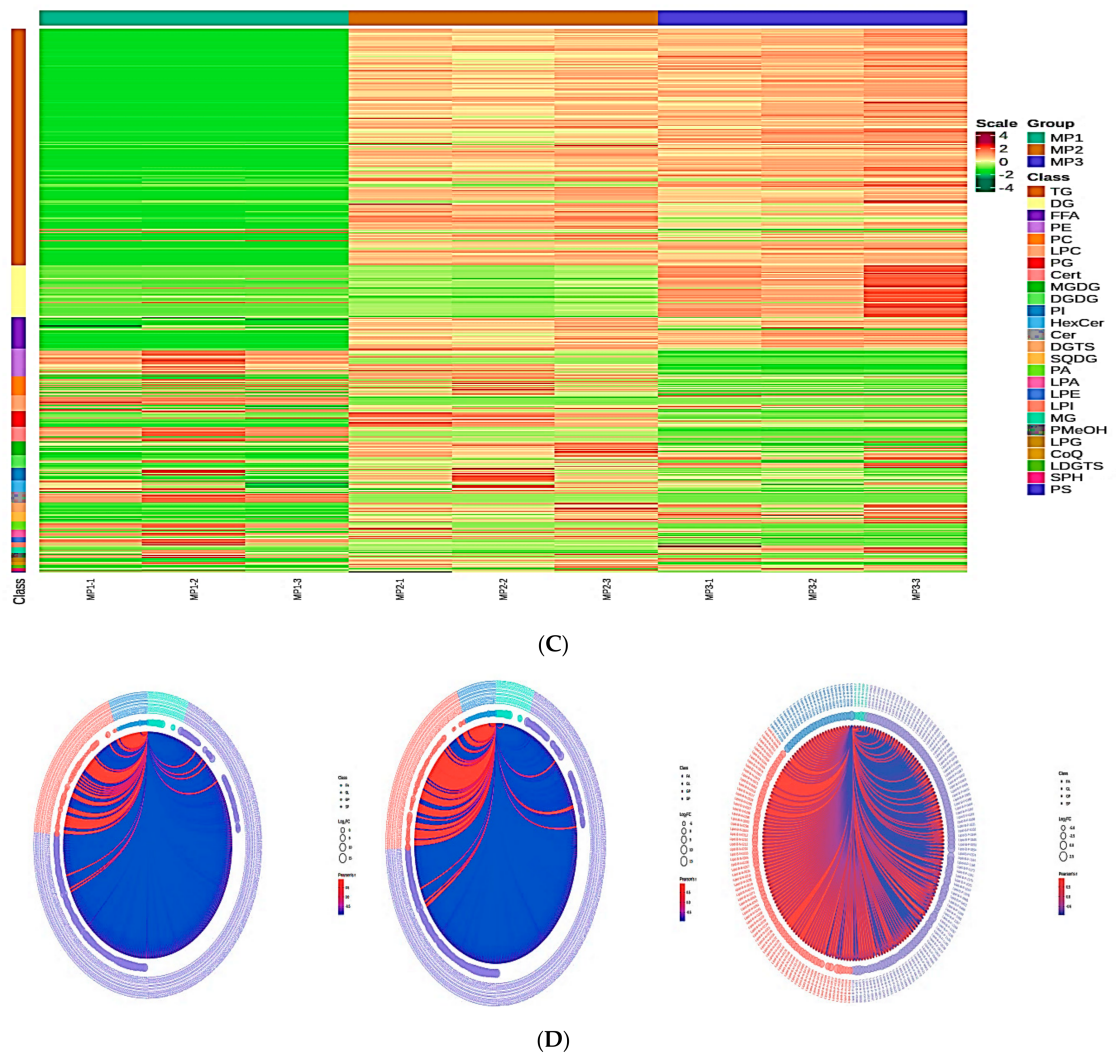
(**A**) Metabolite bar graph showing different phases of metabolism. (**B**) The dynamic distribution of the metabolite content variations. (**C**) Heat map of the metabolite clustering. (**D**) Schematic representation of a differential metabolite chord.

In this study, substantially different lipid metabolites (fold change = 2 or VIP = 1, *p*-value = 0.05) were screened. Based on the magnitude of the log2FC values for the lipid metabolites, a metabolite bar graph was constructed (Figure 5A). To more precisely and graphically illustrate the overall differences in metabolites, the dynamic distribution of the metabolite content differences is shown based on the FC values (Figure 5B). We used unit variance scaling to normalize the raw content of the different metabolites. This made it easier to view the different patterns of the metabolite content and the heat map was constructed (Figure 5C). Pearson correlation analyses were determined to identify the correlation of the differential metabolites and a heat map of the correlations for the differential metabolites was created (Figure S1). The top lipid metabolites of the different phases (MP1, MP2, MP3) were plotted on a violin plot to display the data distribution and probability density based on the VIP values of the metabolites (Figure S2). Figure 5D displays the differential metabolite chord diagram. Plots were automatically generated for differential metabolites with *p <* 0.05.

### 3.6. Transcriptome Analysis of the Oil Palm

The transcriptome profile of the oil palm was examined for the developing oil palm mesocarp at 95, 125, and 185 days after pollination. A Pearson correlation analysis showed that all libraries from the biological replicates were highly related, and thus, the quality of the samples was high enough for gene expression analysis. Pairwise comparisons between three developmental stages were performed and 469 DEGs were identified, with 411, 419, and 193 DEGs found for MP1_vs_MP2, MP1_vs_MP3, and MP2_vs_MP3, respectively (Figure S3). It was noted that the pairwise comparisons, MP1_vs_MP3 had the largest number of DEGs exhibiting significantly up- or downregulated expression.

### 3.7. Kyoto Encyclopedia of Genes and Genomes Pathway Analysis of Differential Lipid Metabolites

In order to better understand the patterns of variations in groups of significant metabolic pathways, KEGG metabolic pathways were clustered to identify the contents of all metabolites in these pathways (Figure S4).

**Figure 6:**
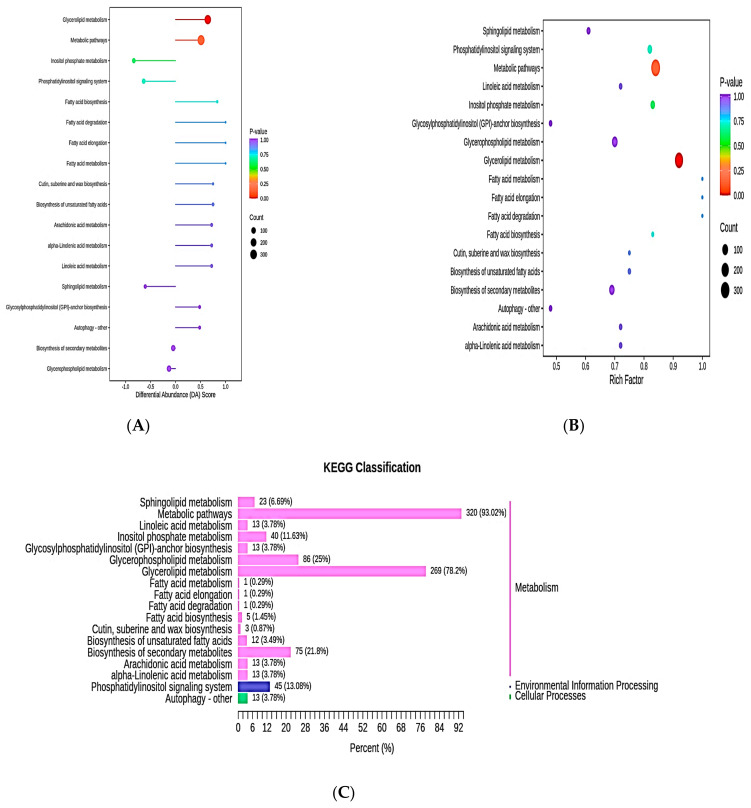
(**A**) Differential abundance score of the metabolic pathways. (**B**) KEGG enrichment map. (**C**) KEGG enrichment analysis of the lipid metabolites.

The differential abundance score (DA score) is found using a metabolic change study based on pathways. The range of the DA score is from −1 to 1. For all identified pathway metabolites, a score of −1 denotes a tendency toward downward regulation, while a score of 1 denotes a tendency toward upward regulation. In order to identify distinct metabolites, we created a KEGG enrichment map (Figure 6A). The richness factor is calculated as the ratio of total metabolites identified by pathway identification of total metabolites differentially expressed in the relevant pathway. A highly significant value signifies a larger accumulation. Figure 6B shows the calculated hypergeometric test *p*-value. In the KEGG enrichment map of the differential metabolites, the horizontal coordinate is the richness factor for each pathway, the vertical coordinate is the pathway name, and the color of the dot represents the *p*-value. The size of each dot indicates the number of differentially enriched metabolites. As shown in Figure 6C, the whole KEGG annotation results of differentially significant metabolites were categorized based on the KEGG pathway.

## 4. Discussion

Lipid metabolism is essential for many biological processes, including the metabolic pathway and intercellular information exchange; thus, it is a crucial part of metabolomics [14]. Targeted lipidomics is a promising tool for understanding how environmental contaminants interact with lipid signaling and metabolic pathways [15]. Very few studies have been carried out to characterize lipid-related enzymes, either directly from palm fruit or from other oil crop tissues. For instance, studies showed that purified mesocarp glycerol 3-phosphate acyltransferase (GPAT) has a strong preference for palmitoyl-CoA [16,17]. An acyl-ACP thioesterase from oil palm has specificities for 14:0-ACP and 16:1-ACP [18]. A kernel DGAT1-1 has a preference for MCFAs [19], and a lipase that affects palm oil quality was characterized [20]. However, the mechanism through which the lipid metabolism of oil palm mesocarp occurs remains elusive. Using a metabolomic approach, we determined what factors contributed to oil yield at the biosynthetic level in oil palms. We also investigated the mesocarp metabolites throughout crucial oil production phases of fruit development in three different levels of genetically related oil palm populations in order to increase oil yield. We conducted a qualitative and quantitative investigation of lipid metabolites in the oil palm using LC-MS/MS equipment, establishing complete profiles for 518 lipids from 26 lipid classes. Different forms of lipids were determined in our study at various phases of fruit kernel development. Most of these lipids were glycerolipids, which included triacylglycerols (TAGs). We used LC-MS to evaluate the fatty acid composition and molecular species in oil palm lipids. Initially, we analyzed TAG, DAG, PC, and PE molecular species during the mesocarp and kernel oil accumulation phases of fruit growth. For commercial seed production, oil was extracted from the mesocarp of mature fruits from the *Elaeis guineensis* species. The most prevalent neutral lipid bodies in seeds are TAGs, which are completely acylated derivatives of glycerol [21]. In oil palm, a proteomic study showed that LDAP accumulation occurs during mesocarp development [22]. Unsaturated TAG levels were found in Paeonia ostii [23] and Brassica napus [24]. The amount of stored TAGs is functionally related to stress reduction and seed germination [25]. Various types of diacylglycerols (DAGs) are present in high abundance among other glycerolipids detected in the developing fruit of oil palm. DAGs are precursors to glycolipids, are rapidly phosphorylated by the enzyme diacylglycerol kinase to produce phosphatidic acid (PA), and act as potential second messengers in plants [26].

Fatty acids are synthesized from acetyl-CoA and FATA and FATB are the two thioesterases responsible for producing common FAs [27]. Palm oil (CPO) and palm kernel oil (PKO) have different FA compositions, with PKO being rich in MCFA and lauric acid, while CPO is extracted from the mesocarp and is rich in palmitic acid and stearic acid [28]. The SAD and thioesterase gene families in oil palm are important for controlling the desaturation and accumulation of storage lipids, but it is important to identify which isoforms are involved. Through the biosynthesis of fatty acids and lipids, the high TAG and DAG concentrations in oil palm fruit mesocarp may help to increase the oil quality. Recently, transcription factor lipid synthesis, photosynthesis, cuticle lipid synthesis, and remodeling were used to improve plant oil quality, while further studies are underway to modify the fatty acid composition and enhance the oil content. Oil palm DGAT1 and DGAT2 were identified using transcriptome data [18]. Aymé et al. [19] reported that DGAT1 causes lauric acid accumulation in palm kernel oil, while DGAT2 increases oil accumulation in the mesocarp during fruit ripening, likely affecting palmitic acid and oleic acid enrichment [18]. The OPLS regression analysis was utilized to identify essential metabolites associated with the ripening process. According to Putri and Fukusaki [29], a multivariate analysis is more efficient at revealing the relationship between the explanatory variable and the response variable. The OPLS regression model has three variables that can be used to measure its quality: Q2, R2X, and R2Y. R2 is the square of the correlation coefficient between the observed and projected values in a regression, and Q2 is a reliable parameter for model predictivity. The R2 and Q2 values should be greater than 0.6 and 0.6, respectively, and three OPLS models were created from metabolites annotated in the M1 vs. M2, M1 vs. M3, and M2 vs. M3 comparisons. These modes were stable and reliable and could be used to identify differentially accumulated metabolites. The hierarchical cluster analysis (HCA) results of samples and metabolites were presented as heatmaps with dendrograms, while Pearson correlation coefficients (PCCs) between samples were calculated using the cor function in R and presented only as heatmaps. For the HCA, the normalized signal intensities of metabolites (unit variance scaling) were visualized as a color spectrum.

Lipidomics profiles were used for the KEGG enrichment analysis. The KEGG Compound Database was used to annotate the identified metabolites, which were then incorporated into the KEGG Pathway Database. The *p*-values for the hypergeometric test were used to establish the importance of the pathways where substantially regulated metabolites had been mapped to an MSEA (metabolite sets enrichment analysis). The findings demonstrated that KEGG pathways were considerably enriched for several lipid metabolites. The biological heat map of clusters from different development stages of oil palm was generated. The heat map depicted the datasets as clustered patterns that showed an overview of the distribution of oil palm lipids that were represented. Further exploration of the enrichment pathways demonstrated that fatty acid metabolism, elongation, and degradation were all associated with the metabolome.

## 5. Conclusions

In this study, lipidomic tools were used to analyze the variability of the lipid composition in oil from palm, as well as to characterize the accumulation of oil during the development of the mesocarp. The extensive nature of metabolism during lipid biosynthesis and accumulation is expressed more by targeted lipidomic analysis in oil palm, which revealed a diverse spectrum of molecular species in each lipid class, as well as rapid accumulation of most lipids in the middle and final phases of storage. The lipidomic analysis showed that it was actively engaged in lipid biosynthesis and the metabolic pathway. Overall, distinctions in patterns and lipid biosynthesis were observed at different stages of fruit development. This study provides insight into the molecular mechanisms regulating lipid production and accumulation, providing practical support for oil palm breeding initiatives, as well as molecular enhancement. However, the current study should allow future work to focus on a variety of applications, including validating oil quality, identifying falsified oils, and enhancing traceability.

## Data Availability

The data presented in this study are available on request from the corresponding author. The data are not publicly available due to privacy.

## Supplementary Materials

The following supporting information can be downloaded from https://www.mdpi.com/article/10.3390/metabo13060727/s1, Figure S1: Pearson correlation analysis; Figure S2: Violin plot of the lipid metabolites; Figure S3: Differentially Expressed Genes (DEGs); Figure S4: Cluster Analysis.
