# Supplementary material for: Lipidomic Profiles of Lipid Biosynthesis in Oil Palm during Fruit Development
Source: Metabolites. 2023 Jun 6;13(6):727. doi: 10.3390/metabo13060727 (PMC10304120; doi:10.3390/metabo13060727)
Supplement: Supplementary file 1 [file metabolites-13-00727-s001.zip › metabolites-2352364-supplementary.pdf]

Supplementry Figure:

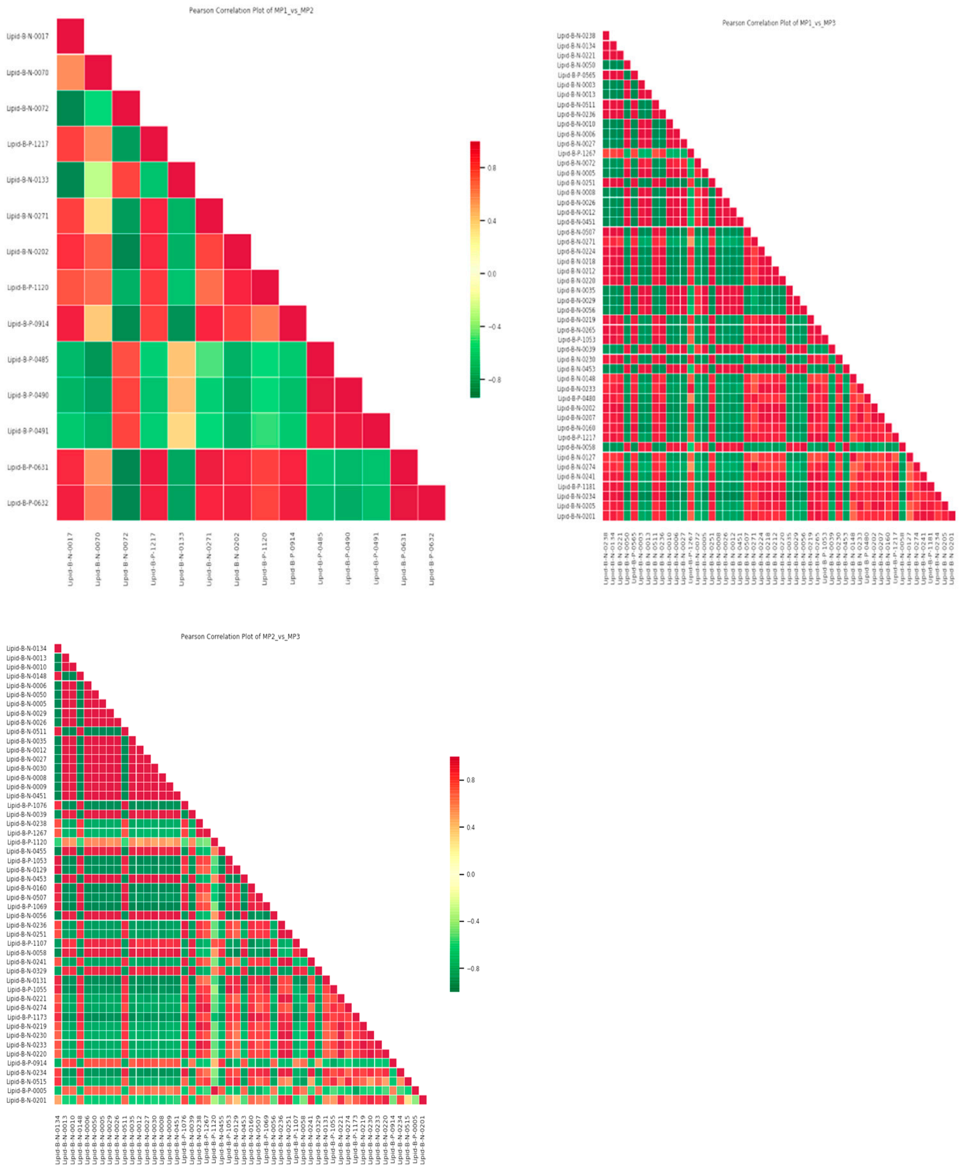

Figure S1: Pearson correlation analysis were determine the correlation of the differential metabolites and created a heat map of the correlation for the differential metabolites

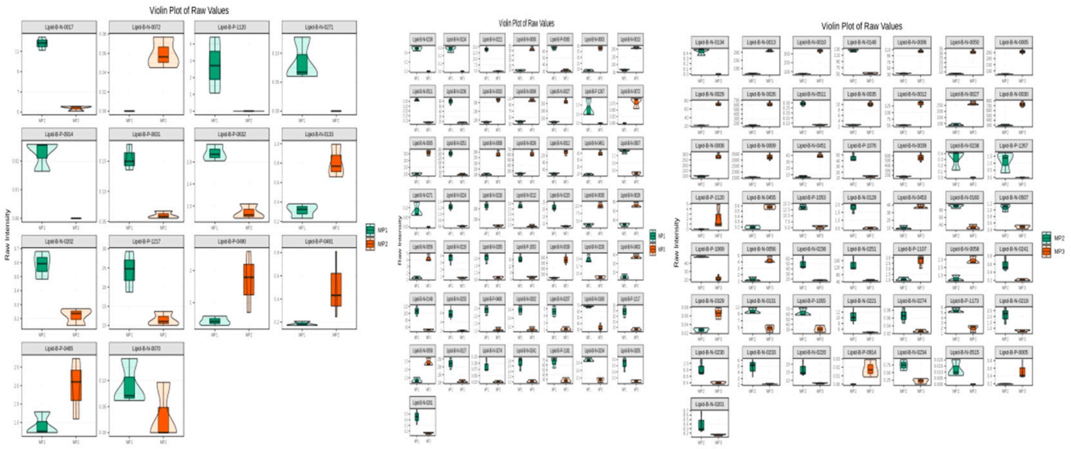

Figure S2: Based on the Variable Importance in Projection (VIP) plot of the metabolites that were showed the data distribution and probability density, and violin plot of the top 50 lipid metabolites was constructed.

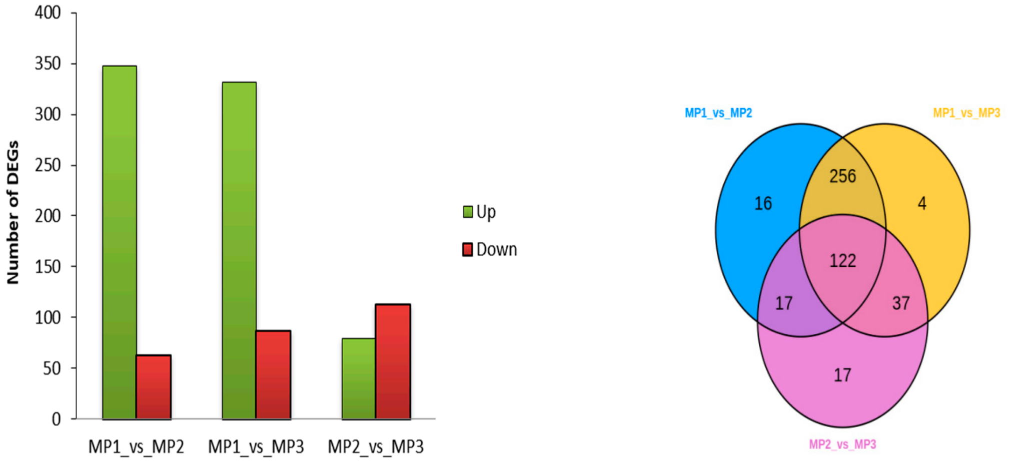

Figure S3: Numbers of differentially expressed genes (DEGs) in comparison with the developing oilpalm fruit between developmental stages (A) and Venn diagram of DEGs among different comparisons (B)

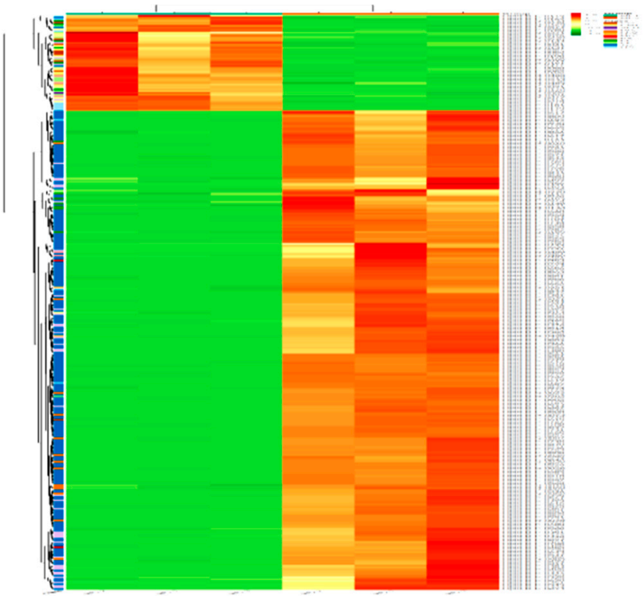

Figure S4: Clustering analysis for different metabolites of KEGG signaling pathway.
